# Standard versus eversion-modified double-staple technique for low colorectal anastomoses after resection of rectal cancer

**DOI:** 10.1007/s00595-020-02174-5

**Published:** 2020-10-31

**Authors:** Giulio Illuminati, Rocco Pasqua, Bruno Perotti, Paolo Urciuoli, Priscilla Nardi, Chiara Fratini, Fabio Carboni, Mario Valle

**Affiliations:** 1 The Department of Surgical Sciences, University of Rome “La Sapienza”, Policlinico Umberto Primo, Viale del Policlinico, 00166 Rome, Italy; 2 The Department of Surgical Oncology, Regina Elena Cancer Institute, Rome, Italy

**Keywords:** Colorectal cancer, Double-stapled colorectal anastomosis, Stapling technique

## Abstract

**Purpose:**

The double-staple technique, performed as either the standard procedure or after eversion of the rectal stump, is a well-established method of performing low colorectal anastomoses following the resection of rectal cancer. Eversion of the tumor-bearing ano-rectal stump was proposed to allow the linear stapler to be fired at a safe distance of clearance from the tumor. We conducted this study to compare the results of the standard versus the eversion-modified double-staple technique.

**Methods:**

The subjects of this retrospective study were 753 consecutive patients who underwent low stapled colorectal anastomosis after resection of rectal cancer. The patients were divided into two groups according to the method of anastomosis used: Group A comprised 165 patients (22%) treated with the modified eversion technique and group B comprised 588 patients (78%) treated with the standard technique. The primary endpoints of the study were postoperative mortality, surgery-related morbidity, the number of sampled lymph nodes in the mesorectum, and late disease-related survival.

**Results:**

Postoperative mortality was 1.2% in group A and 1.7% in group B (*p* = 0.66). Postoperative morbidity was 12% in group A and 11% in group B (*p* = 0.75). The mean number of sampled lymph nodes in the mesorectum was 23 (range 17–27) in group A and 24 (range 19–29) in group B (*p* = 0.06). The 5-year disease-related survival was 73% in group A and 74% in group B (*p* = 0.75).

**Conclusion:**

The standard and eversion-modified double-staple techniques yield comparable results.

## Introduction

Anterior resection of the rectum with complete excision of the mesorectum, performed either as an open surgery or laparoscopically, remains the standard treatment for rectal cancer [1–4]. The double-staple technique [5] is widely accepted and has simplified low colorectal and coloanal anastomoses after anterior resection for cancer. Applying the linear stapler to close the anorectal stump within the abdomen can be difficult or unsafe to ensure a correct clearance distance from the tumor; therefore, everting the anorectal stump bearing the tumor, with extra-anal closure of the stump itself and resection of the tumor, followed by gentle re-positioning of the stump back into the perineum and anastomosis with a circular stapler is a feasible alternative [6]. This modified type of double stapling with the eversion technique has been performed successfully in recent years, with good results [4, 7, 8]. However, there is some concern that the eversion technique may not be optimal for complete resection of the mesorectum without entering the mesorectal fascia. Although a previous study focusing on the long-term results of the eversion technique seems to exclude such concerns [4], a direct comparison between the standard double-staple technique and the modified eversion technique has not been done. A minor but important concern is the possibility that eversion of the anorectal remnant may cause functional neurogenic impairment, resulting in a higher incidence of postoperative neurogenic bladder and fecal incontinence. We conducted this retrospective study to evaluate the outcomes of the two techniques by comparing two homogeneous groups of patients, excluding this technical variable, to validate the assumption that they are oncologically equivalent.

## Materials and methods

Between January 1, 1990 and December 31, 2019, 753 consecutive patients underwent a low, stapled colorectal anastomosis for rectal cancer located 4.4–7.7 cm from the anal verge, at a tertiary academic hospital in Italy and an affiliated, high-volume research hospital dedicated to oncologic surgery. Their clinical records were entered in a database, which was analyzed retrospectively. Informed consent for surgery was obtained from all the patients, whereas institutional review board approval was waived given the retrospective nature of the study. The patients were divided into two groups. Group A, comprised 165 patients (22%) who underwent a modified version of the colorectal anastomosis described by Knight and Griffen [8], consisting of an eversion and extra-anal resection of the rectal stump bearing the tumor, followed by linear suture of the stump itself. The operation was performed as open surgery in 131 patients (79%) and laparoscopically in 34 (21%). Group B comprised 588 patients (78%) who underwent the standard, double-staple technique, performed by open surgery in 399 (68%) and laparoscopically in 189 (32%).

Table 1 summarizes the demography and baseline characteristics of the patients, which were comparable in the two groups. There was no preoperative criterium to select patients to undergo either one of the two techniques. As reported previously [4], the modified technique was used strictly whenever stapled closure of the rectum from the abdomen was considered unsafe intraoperatively, to ensure a safe distance of clearance from the tumor. Therefore, the selection for an eversion, modified technique was made intraoperatively, by the surgeons who decided to proceed with an eversion of the anorectal-stump if they were not confident about achieving clearance from the lower tumor margin by applying the linear stapler intra-abdominally.

**Table 1:** Demography and baseline characteristics of the two groups of patients

Category	Group A	Group B	*p *value
Men	122 (74%)	417 (71%)	0.45
Age, years	67 (38–88)	68 (34–85)	0.14
Body mass, Kg/mq	25 (17–31)	26 (18–32)	0.15
ASA Score III–IV	20 (12%)	88 (15%)	0.36
Anticoagulant/antiplatelet	18 (11%)	70 (12%)	0.73
B-blockers	30 (18%)	94 (16%)	0.50
Current smokers	39 (24%)	129 (22%)	0.64
HTA	58 (35%)	223 (38%)	0.51
CAD	11 (7%)	29 (5%)	0.38
CRI	5 (3%)	17 (3%)	0.83
PAD	5 (3%)	11 (2%)	0.30

*ASA* American Society of Anesthesiologists, *CAD* coronary artery disease, *CRI* chronic renal insufficiency (> 160 mmol/L), *PAD* peripheral arterial disease

**Fig. 1:**
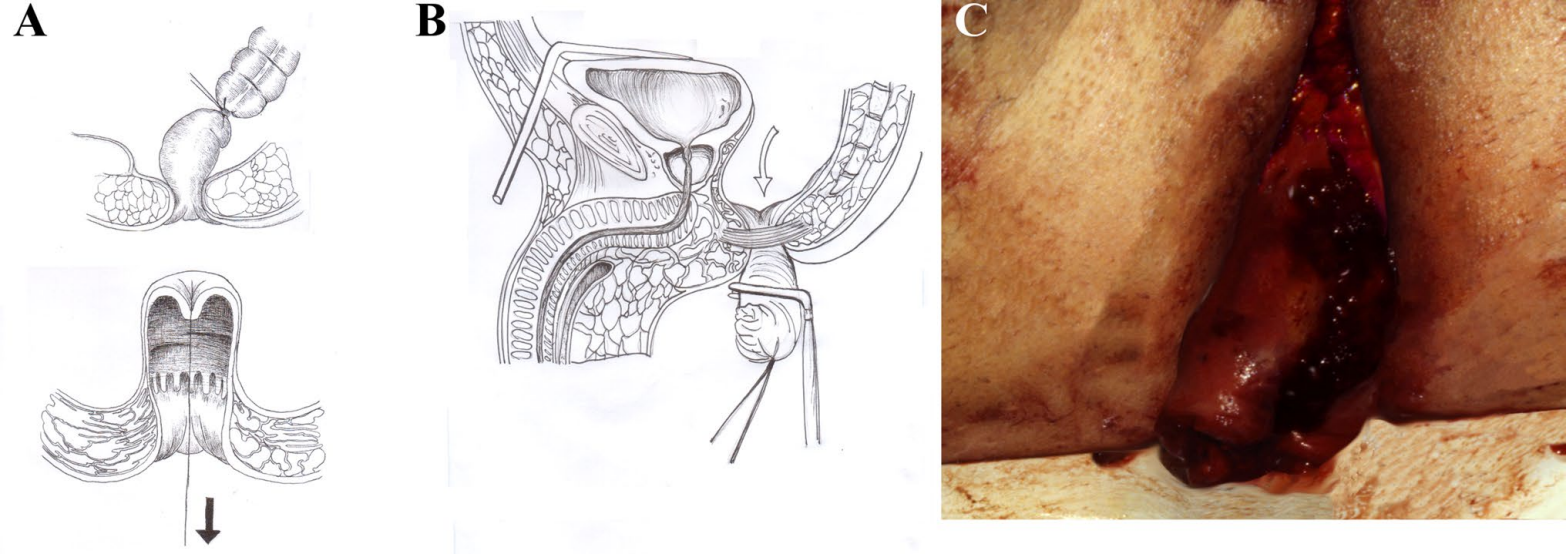
After eversion of the rectal stump with the tumor (**a**), the linear stapler is applied a safe clearance distance from the tumor itself, as shown in the drawing (**b**) and in the intraoperative picture (**c**)

**Fig. 2:**
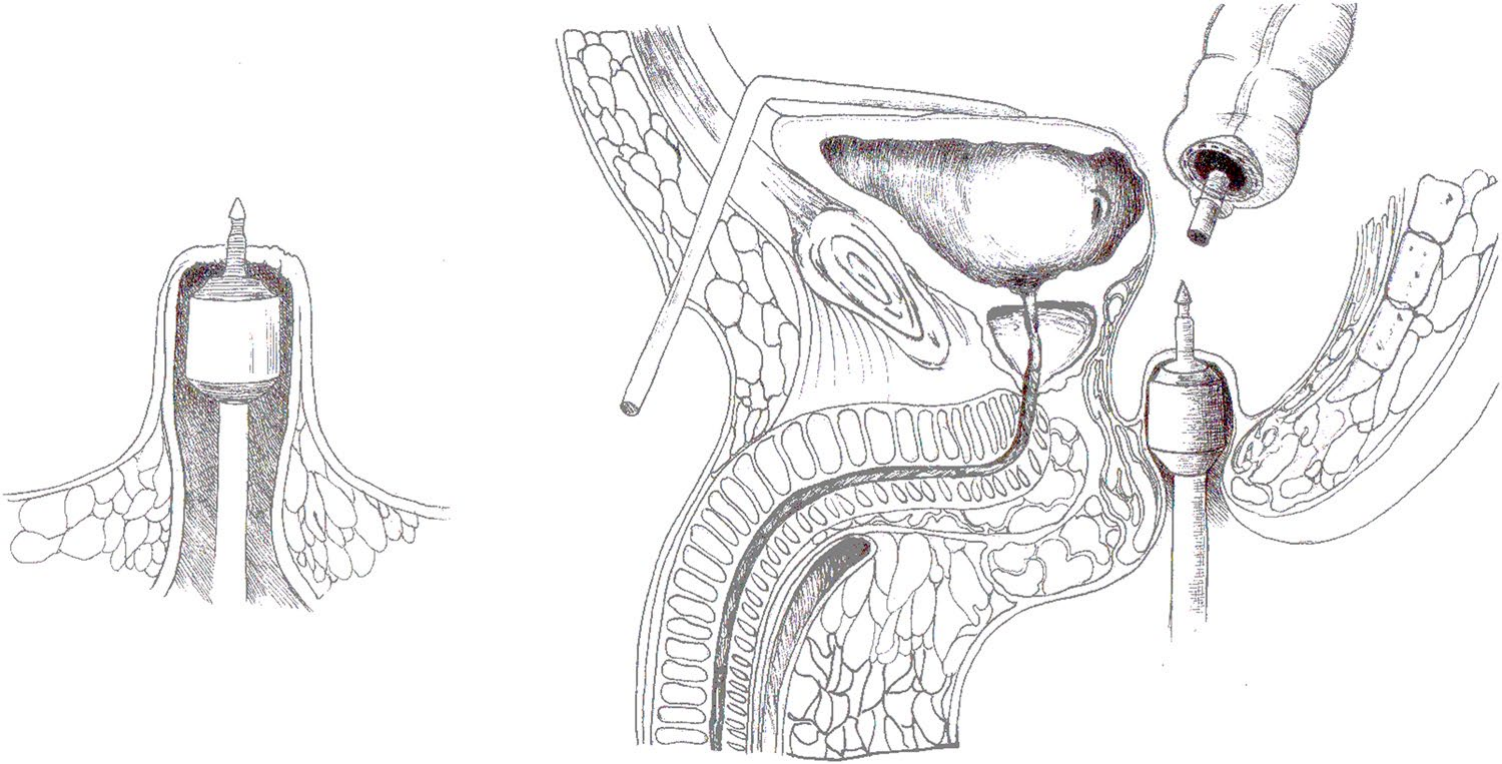
After gently pushing back the closed rectal remnant, a stapled low colorectal/coloanal anastomosis is performed

Briefly, the modified technique involves dividing the rectum a few centimeters above the tumor after complete removal of the mesorectum without entering the mesorectal fascia, pulling the rectal stump out of the anus, applying the linear stapler a safe clearance distance from the tumor, resecting the tumor, pushing the stump back into the pelvis, and performing a low colorectal or coloanal anastomosis with a circular stapler [6] (Figs. 1, 2).

The mean distance of the tumor from the anal verge was 6.2 cm (range 4.4–7.5 cm) in group A and 5.8 cm (range 4.4–7.7 cm) in group B (*p* = 0.14). After surgery, all the patients were referred to the oncology department for adjuvant treatment and follow-up. The mean follow-up period was 53 months (range 3–156 months). No patient was lost to follow-up.

### Primary endpoints

The study’s primary endpoints were postoperative mortality and surgery-related morbidity, the number of sampled lymph nodes in the mesorectum, and late disease- and stage-related survival. Postoperative mortality was defined as any death within 30 days after surgery or during postoperative hospitalization. Postoperative morbidity was considered as any postoperative surgery-related condition either requiring re-operation or prolonging the postoperative stay in the hospital beyond 11 days. The number of sampled lymph nodes was defined as the number of lymph nodes retrieved in the mesorectum at the pathological examination of the surgical specimen. Late disease-related survival was defined as any death beyond 30 days after discharge from hospital, directly related to the progression of the disease. Stage-related survival was defined as any late disease-related death correlated to the stage of the disease at the time of operation.

### Secondary endpoints

The length of distal clearance margin, need for a protective stoma, postoperative length of stay in the hospital, rate of local recurrence, the occurrence of neurogenic bladder, late anastomotic stricture, fecal incontinence, and incisional hernias were considered as secondary endpoints. The distal clearance margin was defined as the length of rectum distal to the disease-free tumor, assessed at the pathological examination of the surgical specimen. Local recurrence was defined as evidence of new tissue growing at the site of surgical resection or anastomosis, evident on CT scan or colonoscopy during follow up and appearing after a previous CT scan or colonoscopy without specific signs of new neoplastic growth. The need for a protective stoma was defined as any stoma performed either at the time of operation or afterwards. Indications for a protective stoma at the time of operation were neo-adjuvant chemo-radiotherapy and eventual intraoperative fecal contamination. Anastomotic leakage was an indication for a protective stoma postoperatively. The postoperative length of stay was defined as the number of days from the day of operation to the day of discharge from hospital. Neurogenic bladder was defined as any postoperative urine overflow incontinence, frequency, urgency, or retention that developed postoperatively. Anastomotic stricture was defined as any decrease in the inner lumen at the anastomotic site at colonoscopy, associated with impairment of defecation. Fecal incontinence was defined as difficulty or inability to control stool leakage postoperatively. Incisional hernia was defined as any surgical incision site hernia requiring surgical repair.

### Statistical analysis

We compared the clinical variables of the endpoints with a Chi-square test for categorical variables and a Student’s *t* test for continuous variables. Survival and rates of local recurrence are expressed by a life-table analysis [9]. Differences were considered significant for a *p* value < 0.05.

## Results

The T value and stage of the disease at the time of operation were comparable in the two groups, according to the Union for International Cancer Control (UICC) classification (Tables 2, 3) [10]. Thirty-four patients (21%) in group A and 189 (32%) in group B underwent laparoscopic resection (*p* = 0.004). Hepatic metastases were found at the time of surgery in 11 patients (7%) from group A and 37 patients (6%) from group B (*p* = 0.86). The mean distance of the tumor from the anal verge was 6.2 cm (range from 4.4 to − 7.5 cm) in group A and 5.8 cm (range from 4.4 to − 7.7 cm) in group B (*p* = 0.14). One group B patient underwent simultaneous open resection of a concomitant aneurysm of an aberrant splenic artery arising from the superior mesenteric artery [11]. The mean operation time was 235 min in group A (range 220–330 min) and 231 min in Group B (range 200–310 min) (*p* = 0.07). The average intraoperative blood loss was 370 ml in group A (range 310–620 ml) and 365 ml in group B (range 300–600 ml) (*p* = 0.20). Ninety-five patients (58%) in group A and 341 (58%) in group B (*p* = 0.92) received preoperative neoadjuvant treatment for a T3 – T4 tumor, whereas 133 patients (81%) in group A and 463 (79%) in group B (*p* = 0.60) received postoperative adjuvant treatment for stage II or III disease. Of the 95 patients who received neo-adjuvant treatment in group A, 18 (19%) underwent subsequent laparoscopic resection and 77 (81%) underwent open resection, whereas of the 341 patients who received neoadjuvant treatment in group B, 102 (30%) underwent laparoscopic intervention and 239 (70%) underwent open intervention (*p* = 0.03). The standard protocol for neo-adjuvant treatment consisted of 30–50 Gy radiation therapy associated with five fluorouracil plus folinic acid for 5 weeks, whereas adjuvant treatment consisted of six cycles of five fluorouracil and folinic acid.

**Table 2:** *T* value at the time of operation according to the UICC classification [10]

*T* value	Group A	Group B	*p *value
T1	15 (9%)	41 (7%)	0.36
T2	55 (33%)	206 (35%)	0.69
T3	77 (47%)	294 (50%)	0.45
T4	18 (11%)	47 (8%)	0.24
Tot	165 (100%)	588 (100%)	–

**Table 3:** Disease stage at the time of operation according to the UICC classification [10]

Stage	Group A	Group B	*p* value
I	25 (15%)	96 (16%)	0.72
II	44 (27%)	164 (28%)	0.75
III	89 (54%)	299 (51%)	0.48
IV	7 (4%)	29 (5%)	0.71
Tot	165 (100%)	588 (100%)	–

### Primary endpoints

Two patients (1.2%) from group A died in the postoperative period: one of myocardial infarction and one of multiorgan system failure. Ten patients (1.7%) from group B died in the postoperative period: five of myocardial infarction, three of sepsis, and two of adult respiratory distress syndrome. This difference was not significant (*p* = 0.66). Overall, there was 12% postoperative morbidity in group A and 11% in group B (*p* = 0.75), with 7 anastomotic leaks, 9 wound infections, 2 ureteral lesions, and 2 peristomal abscesses in group A; and 34 wound infections, 26 anastomotic leaks, and 6 stoma abscesses in group B.

**Fig. 3:**
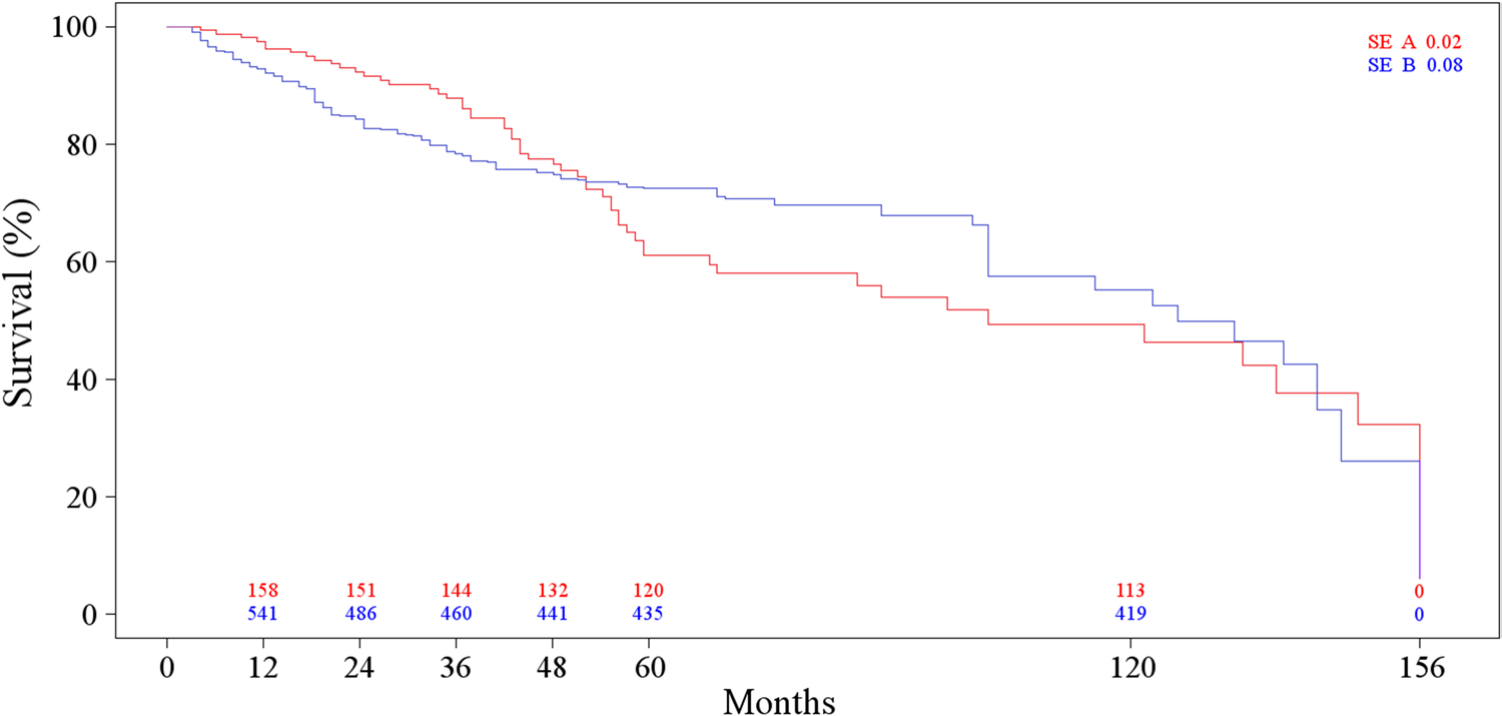
Long-term disease-related survival. The numbers at the bottom represent the number of patients at risk at each time interval. *SE* standard error

The mean number of sampled lymph nodes in the mesorectum was 23 (range 17–27) in group A and 24 (range 19–29) in group B (*p* = 0.06). At 5 years, disease-related survival was 73% in group A and 74% in group B (*p* = 0.75) (Fig. 3). Stage-related survival at 5 years in group A was 92% for stage I, 86% for stage II, 40% for stage III, and 14% for stage IV, whereas in group B, it was 94% for stage I (*p* = 0.66), 84% for stage II (*p* = 0.91), 38% for stage III (*p* = 0.91), and 10% for stage IV (*p* = 0.88). None of these differences were significant. Table 4 summarizes the primary endpoints.

**Table 4:** Primary endpoints of the study

Endpoint	Group A	Group B	*p* value
Mortality	2 (1.2%)	10 (1.7%)	0.66
Morbidity	20 (12%)	66 (11%)	0.75
Sampled lymphnodes in the mesorectum	23	24	0.06
Survival (5 years)	120 (73%)	435 (74%)	0.75

### Secondary endpoints

The mean distal clearance from the tumor was 2.3 cm (range 2.0–4.4 cm) in group A and 2.7 cm (range 2.0–4.8 cm) in group B (*p* = 0.14). A protective stoma was needed by 123 patients (75%) in group A and 423 patients (72%) in group B (*p* = 0.51). Of those patients, 109 (87%) in group A and 359 (85%) in group B underwent subsequent stoma closure with a mean delay of 90 days (range 31–451 days) and 89 days (range 27–440 days), respectively (*p* = 0.08). The mean postoperative length of stay in hospital was 10 days (range 7–28 days) in group A and 9 days (range 7–31 days) in group B ( *p* = 0.12). Five patients (3.0%) in group A and 12 patients (2.0%) in group B (*p* = 0.45) suffered neurogenic bladder postoperatively. Eight patients (4.9%) in group A and 26 patients (4.4) in group B had late anastomotic stricture requiring endoscopic dilatation (*p* = 0.82), whereas 6 patients (3.6%) in group A and 12 patients (2.0%) in group B (*p* = 0.24) suffered late fecal incontinence. Incisional hernia requiring surgical repair developed in 7 patients (4.2%) in group A and 30 (5.1%) in group B (*p* = 0.65). Local recurrence of the tumor developed in nine patients (5.5%) in group A and 25 patients (4.3%) in group B. This difference was not significant (*p* = 0.51). Table 5 summarizes the secondary endpoints.

**Table 5:** Secondary endpoints of the study

Endpoint	Group A	Group B	*p *value
Mean length of distal clearance	2.3 cm (2.0–4.4 cm)	2.7 cm (2.0–4.8 cm)	0.14
Protection stoma	123 (75%)	423 (72%)	0.51
Postoperative length of stay	10 (7–28)	9 (7–31)	0.12
Neurogenic bladder	5 (3%)	12 (2.0%)	0.45
Fecal incontinence	6 (3.6%)	12 (2.0%)	0.24
Anastomotic stricture	8 (4.9%)	26 (4.4%)	0.82
Incisional hernia	7 (4.2%)	30 (5.1%)	0.65
Local recurrence	9 (5.5%)	25 (4.3%)	0.51

## Discussion

The results of this study show that the modified version of the double-stapling technique after eversion for stapled low colorectal or coloanal anastomosis, extra-anal resection of the tumor and stapled closure of the anorectal stump, performed with open [4, 6] or laparoscopic [1] surgery, is comparable to the standard double-stapling technique. This equivalence is important when considering the outcomes of both the techniques, such as postoperative mortality/morbidity, the number of sampled lymph nodes in the mesorectum, long-term survival, and local recurrence rates. The major concern about the modified technique, since its first description, is that it may impair correct and complete resection of the mesorectum at the time of sectioning the rectum above the tumor for eversion. Although it has been shown that complete resection of the mesorectum en bloc with the everted specimen can be performed with good oncologic results [4, 7, 8], validation of this technique with a homogeneous group of patients undergoing the classic, standard double-stapling technique for the same indications, same stage of disease, and same tumor characteristics was mandatory. Our retrospective comparison of the two patient groups with superposable variables, except for the technique, seems to validate the former assumption of equivalent results of the two techniques. In addition to postoperative mortality and morbidity, under a strictly technical analysis, this is underscored by the comparable number of lymph nodes sampled in the mesorectum, the incidence of anastomotic leaks and fistulas, and local recurrence rates. The results of this study are also comparable to those reported in the literature [2, 3, 12–22] and the relatively high incidence of protective stomas in the present series is probably explained by the large number of patients undergoing neo-adjuvant treatment in both study groups. In other words, everting the tumor-bearing anorectal remnant to allow tumor resection and closure of the anorectal remnant does not seem to increase the risk of fistulas or local recurrence or to impair survival. One further element supporting this assumption is the fact that superposable results with the two techniques can be obtained when both are performed laparoscopically. The laparoscopic feasibility of the modified technique has been reported previously [1], but a direct comparison of two series, including a sufficient number of laparoscopic resections was warranted. In this study, two series that included laparoscopic access were compared and yielded results that were comparable not only to each other, but also to those of standard double-stapling resections performed with the double-stapling technique reported in the literature [3]. However, the significant prevalence of laparoscopic interventions in group B may have biased the results. In fact, there may be even fewer indications for using the modified technique with open surgery than with the laparoscopic approach when considering applying the linear stapler at a safe clearance distance from the tumor in obese patients, whereas a narrow pelvis may be easier to access laparoscopically. Nevertheless, there may be instances necessitating the use of the modified technique when performing laparoscopic resections [1, 23].

The main concern with the modified technique is the risk of incomplete resection of the mesorectum and damage to the mesorectal fascia. The results of the current study support the hypothesis of substantial equivalence in terms of oncologic standards of the eversion modified technique versus the standard double-staple technique [2, 4, 13, 18, 24–27]. This study also shows that everting the anorectal remnant will not increase the risk of neurologic impairment, such as a neurogenic bladder or fecal incontinence, compared with the standard technique, as the incidence of these complications remained very low overall and comparable between the two techniques. This validation does not mean that indications for the modified technique should be extended. On the contrary, as previously reported [1, 4, 6], they should be strictly limited to special intraoperative settings hindering a safe distal application of the linear stapler with a sound clearance distance from the tumor within the abdomen, in open and laparoscopic surgery.

The main limitation of this study was its retrospective nature and the substantial difference in the number of patients in the two compared groups. Nonetheless, the groups were sufficiently homogeneous, and data were recorded and verified objectively. Given the special setting of the application of the modified technique, it is unlikely that comparable patients could be entered into a preoperatively randomized study to prospectively compare the two techniques. Finally, there was a significantly higher percentage of laparoscopic interventions in group B, in conjunction with a higher number of patients undergoing laparoscopic resection after neoadjuvant treatment, which may not make the two groups of patients perfectly superposable, although it should not have biased the results significantly. The prevalence of laparoscopic resections in group B is related to the long span of the study, with more laparoscopic interventions being performed in recent years, with progressive amelioration of technical skills in performing the classical technique.

In conclusion, this study supports the current concensus that when performing low colorectal resections and anastomoses for cancer, the standard and eversion-modified double-stapling techniques yield superposable technical and oncological results. Concerns about limitations of this technique on the curative aspects of mesorectal resection are not justified and should not hinder its use when intraoperative situations indicate a shift to eversion of the anorectal remnant to improve the distal clearance margin and safety of the low colorectal or coloanal anastomosis.
